# Royal Jelly Increases Hematopoietic Stem Cells in Peripheral Blood: A Double-Blind, Placebo-Controlled, Randomized Trial in Healthy Subjects

**DOI:** 10.1155/2023/7665515

**Published:** 2023-01-13

**Authors:** Hideto Okamoto, Akio Ohkuma, Mitsuhiko Kawaguchi, Norihiro Shigematsu, Nobuaki Okumura

**Affiliations:** ^1^Institute for Bee Products & Health Science, R&D Department, Yamada Bee Company, Inc., Okayama, Japan; ^2^Research Center for Immunology & Analysis, Inc., Okayama, Japan; ^3^Kawaguchi Internal Medicine Clinic, Kawaguchi, Japan

## Abstract

**Objectives:**

Royal jelly (RJ), produced by honeybees, influences stem cell functions, such as pluripotency maintenance of mouse embryonic stem cells and prevention of aging-related muscle stem cell functional deterioration. Thus, we hypothesized that RJ administration has various health-promoting effects based on stem cells. However, its effects are unknown in humans. In this study, we have attempted for the first time to clarify whether the administration of RJ in humans affects stem cells.

**Materials and Methods:**

This randomized, double-blind, placebo-controlled study was performed on healthy subjects (*n* = 90) who received protease-treated RJ at a dose of 1200 mg/day or placebo daily for four weeks. Also, the participants with a low number of hematopoietic stem cells (HSCs) in peripheral blood were preferentially selected. HSC counts, endothelial progenitor cell (EPC) counts, blood cell counts in peripheral blood, cytokines in serum, and physical conditions were evaluated. *Results and Conclusion*. Eligible data from 86 subjects (placebo: 42, RJ: 44) who completed the study were analyzed. There were no significant differences between the two groups regarding the changes in peripheral HSC count (*p*=0.103), while diastolic blood pressure showed a significant improvement in the RJ group compared to that in the placebo group (*p*=0.032). The subgroup analysis excluded 14 subjects who complained of cold symptoms at baseline or within five days of the four-week study. The changes in the HSC populations were significantly higher in the RJ group than those in the placebo group (*p*=0.042). No adverse effects were observed in any of the groups. These results suggest that RJ administration affected the peripheral HSC count and may influence stem cell functions. Further research is needed to reveal the various health-promoting benefits of RJ based on stem cells.

## 1. Introduction

As the average human lifespan has increased worldwide due to improvements in healthcare and public health, the number of elderly people complaining of age-associated symptoms such as immunosenescence and anemia is increasing [1, 2]. The decreased quality of life due to age-related conditions causes the separation of health span and lifespan [3]. Aging is the inevitable time-dependent decline in physical functions and causes altered intercellular communication, genomic instability, telomere attrition, epigenetic alterations, loss of proteostasis, deregulated nutrient-sensing, mitochondrial dysfunction, and cellular senescence. Moreover, previous reports suggest that aging influences stem cell exhaustion and senescence [4].

Stem cells have the capability of self-renewal and differentiation. Embryonic stem cells can maintain pluripotency even if they proliferate for a long period, while somatic stem cells can differentiate only into limited tissues. Hematopoietic stem cells (HSCs), a type of somatic stem cell, produce all blood cell types, including red blood cells, platelets, myeloid cells, and lymphoid cells. They play an important role in maintaining homeostasis in immunity and vascular function [5, 6]. However, aging causes a reduction in the number of circulating HSCs and an increase in age-related markers such as senescence-associated beta-galactosidase, p53, and telomere attrition. This causes a disturbance in homeostasis and a rise in the risk of anemia and infection [7]. Ongoing studies are investigating whether the aged HSC function can be restored and may lead to the treatment of age-related immune disorders and the improvement of quality of life in the elderly [8].

Royal jelly (RJ) is a yellowish-white, creamy, acidic secretion from the cephalic glands of honeybees (*Apis mellifera*). RJ is an essential nutrient for the development of queen honeybees and is involved in longevity and fertility. Previous studies demonstrated that RJ is a functional food that is expected to show anti-aging effects [9]. For example, RJ administration showed improvement in the vascular endothelial function [10] and alleviation of menopausal symptoms [11].

Some reports show that RJ has effects on stem cells. RJ administration promoted the regeneration of injured muscle in aged mice via the IGF1-Akt signal pathway in satellite cells (muscle stem cells) [12]. Moreover, RJ delayed the age-dependent deregulation of the muscle satellite cell marker Pax7 [13]. Additionally, royalactin, a major compound of RJ, maintained mouse embryonic stem cell pluripotency [14]. These results demonstrate that RJ may function as a nutrient that activates stem cells. Therefore, we hypothesized that the health benefits of RJ are based on stem cell. To test this hypothesis, we focused on HSCs relevant to age symptoms, such as immune decline and anemia, and arteriosclerosis. It is also an advantage that the protocol of HSC measurement was established by the International Society of Hematotherapy and Graft Engineering (ISHAGE) guidelines [15]. For the abovementioned reasons, we aimed, for the first time, to clarify whether the administration of RJ influences stem cell function in a human clinical study.

## 2. Methods and Materials

### 2.1. Participants

In this study, we recruited subjects with a low HSC count. We believe participants with a low concentration of HSCs have a weakened immune system because children with chronic neutropenia have a low number of hematopoietic progenitor cells (HPCs) and their immune system degenerate [16]. Furthermore, we aimed to confirm whether the number of HSCs decreases with aging, as previously reported [7]. To achieve these aims, it was necessary to recruit many participants during screening. Therefore, we chose healthy subjects, older than 20 years, from the Research Center for Immunology & Analysis, Inc. (Okayama, Japan). The exclusion criteria were as follows:

1. Individuals with a current food allergy or asthma, or a history thereof
2. Pregnant or breastfeeding individuals or individuals who were planning a pregnancy during this study
3. Individuals who took part in another clinical study within 12 weeks before the intake of the test food
4. Individuals who underwent a major surgical procedure of the gastrointestinal tract, such as gastrectomy, gastrointestinal suture, or intestinal resection
5. Individuals with a current serious illness such as liver disease, kidney disease, heart disease, diabetes, rheumatoid arthritis, or autoimmune disorders, such as systemic lupus erythematosus, or a history thereof
6. Individuals who receive medical treatment such as hospital visits or prescription drugs
7. Individuals who experience difficulty ingesting the test food as instructed or staying still until the end of the test
8. Individuals who regularly use dietary supplements and health foods (including foods with functional claims) that may affect the outcome of this study
9. Based on the subject background questionnaire, those working night shift or who lead an extremely irregular lifestyle regarding eating and sleeping
10. Individuals who had given a blood sample above 200 mL within four weeks or 400 mL within three months before the start of this study
11. Individuals with daily alcohol consumption, e.g., over 500 mL of beer per day
12. Smokers
13. Individuals with a body mass index (BMI) lower than 18.5 and higher than 30

### 2.2. Study Design

The present study was performed following the principles of the Declaration of Helsinki (revised at the 64th WMA General Assembly, Fortaleza, Brazil, October 2013) and Ethical Guidelines for Medical and Health Research Involving Human Subjects (Ministry of Health, Labour, and Welfare, Japan, 2017). Approval was obtained from Shiba Palace Clinic Ethics Review Committee (Approval No. 146370_rn-29647). This protocol was registered at the University Hospital Medical Information Network Clinical Trials Registry (UMIN-CTR, https://www.umin.ac.jp/) on January 8, 2021 as UMIN000042951. All participants provided written informed consent before participation.

This study design was conducted in a double-blind, placebo-controlled, randomized clinical trial and included 90 subjects that were randomly assigned to two groups and distributed equally based upon their HSC count in peripheral blood, age, and gender.

This was the first study to investigate the effect of RJ intake on stem cell function using the number of HSCs. However, we conducted a 4-week RJ administration pre-test on 6 healthy subjects (data not shown), and we expected an evaluated sample size of 45 participants per group to provide 80% power (two-sided, *α* = 0.05) to detect a 25% difference in the change of HSC count in peripheral blood, after considering a 10% dropout rate based on the preliminary tests. A power analysis was conducted via JMP software version 5.1 (Cary, NC, USA) to calculate the sample size.

The test food for four weeks was packed in opaque packages by an external individual and was labeled using a two-digit random number code. The codes were not released until the statistical analysis was complete. Therefore, allocation concealment was achieved, and both researchers and subjects were blinded to group allocation.

### 2.3. Test Food

The RJ tablets contained protease-treated royal jelly (pRJ), developed by treating RJ with alkaline proteases to eliminate allergens [17]. The pRJ (YRP-M-201007-1, YRP-M-201027-1) powder was obtained from Yamada Bee Company, Inc. (Okayama, Japan). It was standardized to include a minimum of 3.5% trans-10-hydroxy-2-decenoic acid and a minimum of 0.6% 10-hydroxydecanoic acid. The RJ tablets contained 200 mg of pRJ and composed the following nutritional content (energy: 8.7 kcal; moisture: 0.1 g; proteins: 0.5 g; lipids: 0.1 g; carbohydrates: 1.5 g; ash: 0.2 g; salt equivalent: 25.1 mg). For placebo preparations, starch was used instead of pRJ and adjusted to have nutritional composition similar to pRJ supplements. The pRJ and placebo tablets had an identical appearance so that they were indistinguishable and were both administered six per day for four weeks. Both test foods were produced by Supplement Japan Co., Ltd. (Tokyo, Japan).

### 2.4. Blood Cell Count in Peripheral Blood and Blood Parameters

To assess the effects of RJ intake on stem cells, the primary end point was the HSC count in peripheral blood. In addition, the secondary end points were the number of endothelial progenitor cells (EPCs) relevant to endothelial function, and the other end points were the number of lymphoid cells (B cells, NK cells, T cells, CD4^+^T cells, CD8^+^T cells, and CD8^+^CD28^+^T cells), myeloid cells (monocytes, neutrophils, eosinophils, and basophils), white blood cells, red blood cells, and platelets in peripheral blood. Furthermore, hemoglobin, hematocrit, mean corpuscular volume (MCV), mean corpuscular hemoglobin (MCH), and mean corpuscular hemoglobin concentration (MCHC) were measured as blood parameters. The measurement protocol of HSC count in peripheral blood has been established by the International Society of Hematotherapy and Graft Engineering (ISHAGE) guidelines [15].

Blood samples were drawn into vacutainers containing EDTA-2Na or EDTA-2K for anticoagulation (Terumo Corporation, Tokyo, Japan). Fluorochrome-conjugated anti-human monoclonal antibodies were obtained from Miltenyi Biotec (Bergisch Gladbach, Germany) and BD Biosciences (San Jose, CA, USA). The antibodies are listed in Supplementary Table 1. For staining of HSCs and EPCs, 100 *µ*L of anticoagulated peripheral blood was stained with surface antibodies for 20 min at 25°C in the dark before lysis with Lysing Buffer (BD Biosciences) for 10 min in the dark. Further, peripheral blood mononuclear cells (PBMCs) were isolated by density gradient centrifugation according to standard protocol. For the investigation of lymphoid cell subpopulations, PBMC surface staining was performed for 30 min at room temperature in the dark. All samples were measured using MACSQuant Analyzer 10 (Miltenyi Biotec, Bergisch Gladbach, Germany) using two different panels. The first panel (Supplementary Figure 1) calculated the HSC count and EPC count following the ISHAGE guidelines [15]. The second panel (Supplementary Figure 2) quantified the lymphoid cell count. All acquired data files were analyzed using MACSQuantify™ Software version 2.13 (Miltenyi Biotec, Bergisch Gladbach, Germany). Myeloid cells, white blood cells, red blood cells, platelet, MCV, MCH, and MCHC were measured by BML, Inc. (Tokyo, Japan).

### 2.5. Cytokine Analysis

To examine stem cell mobilization from bone marrow, which is one of the mechanisms to increase HSCs in peripheral blood, C-X-C motif chemokine 12 (CXCL12) was measured using U-PLEX Biomarker Group 1 (human) Multiplex Assays (Meso Scale Diagnostics, Rockville, MD, USA). Similarly, the macrophage colony-stimulating factor (M-CSF) related to endothelial function was determined.

### 2.6. Anthropometry and Questionnaire Evaluation of Physical Conditions

Body weight, height, systolic and diastolic blood pressure, and pulse rate were measured. To investigate the effects of the intake of RJ tablets on a physical condition during interventional trials, questionnaires were administered that related to fatigue, sleep quality, and anemia symptoms. Fatigue was evaluated by the Chalder Fatigue Scale (CFS). The CFS is a self-administered questionnaire with 14 lists scored against a four-pointLikert-type response scale. The total symptom score was a simple additive measure with a score from 0 to 42 [18]. To assess sleep quality, we used the Pittsburgh Sleep Quality Index Japanese Version (PSQI-J). The PSQI-J is a self-rating questionnaire consisting of 18 questions. The self-rated items of the PSQI include seven component scores (range of subscale scores, 0–3): sleep quality, sleep latency, sleep duration, sleep efficiency, sleep disturbance, use of sleeping medication, and daytime dysfunction. The total of these seven component scores yields one global score of subjective sleep quality (range, 0–21) [19]. To analyze anemia symptoms, the participants assessed their degree of anemia with a 100-mm visual analog scale (VAS). The details of the contents are shown in Supplementary Figure 3.

### 2.7. Statistical Analyses

All values indicate the mean ± standard deviation (SD). HSC count, EPC count, all blood cell counts, the concentration of CXCL12 and M-CSF, blood pressure, pulse rate, and VAS of anemia symptoms were compared between the RJ and placebo groups using the Student's *t*-test. The intragroup comparison between the baseline and post-administration measurements after four weeks was assessed by a paired *t*-test. Moreover, a Wilcoxon rank-sum test was performed to compare the score of CFS and PSQI-J between the two groups. Assessments of differences within the groups were performed by the Wilcoxon signed-rank test. Furthermore, Pearson's correlation coefficient was used to investigate the relationship between the HSC count in the peripheral blood and age or BMI at the screening test. A value of *p* < 0.05 was considered statistically significant. Chi-squared test was also used to compare qualitative variables, like gender in both groups. Statistical analyses were conducted using GraphPad Prism version 7 (GraphPad Software, San Diego, CA, USA).

## 3. Results

### 3.1. Clinical Characteristics of the Participants

**Figure 1:**
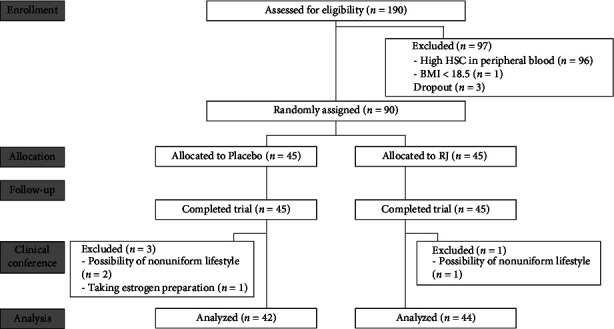
Flow diagram of the study.

The flowchart of the study and recruitment process is shown in Figure 1. Since the primary end point was HSC count in peripheral blood, it was measured during the screening test so that participants with a low number of HSCs in peripheral blood can be preferentially selected. A total of 187 subjects participated in the screening test, with 3 subjects dropped out for personal reasons. Further, we focused on age and BMI and confirmed the correlation to investigate whether the subjects recruited in this study were a universal population. We found a reverse correlation between the HSC count and age (*r* = −0.283, *p* < 0.001). While the number of HSCs in peripheral blood increased with the BMI, no correlation with the BMI was found because a BMI of less than 18.5 and over 30 was set as an exclusion criterion (Supplementary Figure 4). Previous studies reported low circulating HSC numbers in elderly individuals and that obesity promotes the mobilization of CD34^+^ progenitor cells [7, 20]. Therefore, it is probable that this subject population was a universal population.

Of the initial 190 participants, 90 healthy subjects met the eligibility criteria. These 90 participants were randomly assigned to one of two groups, which showed no significant differences in HSC count, age, and gender (Table 1). The 4-week trial was completed by all participants. Four participants were excluded from the analysis (three from the placebo and one from the RJ group). The possibility of nonuniform lifestyle (*n* = 3) and taking an estrogen preparation (*n* = 1) were the reasons for exclusion. Overall, the total number of subjects in the analysis was 86. No serious adverse effects were reported by participants following ingestion of the test food.

The baseline characteristics are presented in Table 2. Although MCV levels were significantly higher in the RJ group than in the placebo group, they were within the normal range (*p* = 0.013). No significant differences between the two groups in terms of age, BMI, HSC count, myeloid cell count, lymphoid cell count, and hematology parameters, except for MCV, were found.

### 3.2. Effect of RJ Intake on HSC Count

The results are tabulated in Table 2. No differences were observed in the changes of HSC count between the two groups. After four weeks of daily tablet intake, the placebo group displayed a lower number of HSCs compared with the baseline, but it was not statistically significant (*p*=0.065). The cell number in the RJ group showed no significant changes between the baseline and post-administration after four weeks.

### 3.3. Effect of RJ Intake on CXCL12

Compared to the placebo group, the RJ group showed no significant difference in CXCL12 levels (Table 3). However, both groups exhibited a significantly higher concentration in comparison with the baseline after four weeks (*p* < 0.001).

### 3.4. Effect of RJ Intake on Vascular Endothelial Function, Blood Pressure, and Pulse Rate

Table 4 presents the number of EPCs in peripheral blood, M-CSF levels, blood pressure, and pulse rate measured. Diastolic blood pressure levels showed significant changes in the RJ group compared to those in the placebo group (*p*=0.032). While the change in systolic blood pressure in the RJ group was lower than that in the placebo group, it was not statistically significant (*p*=0.055). In the RJ group, the systolic and diastolic blood pressure showed a significant improvement after four weeks compared with the baseline (*p* < 0.001 and *p*=0.013, respectively). However, no differences were observed in EPC counts, M-CSF levels, and pulse rate between the two groups. After four weeks of daily tablet intake, the placebo group displayed lower numbers of EPCs compared with the baseline, but it was not statistically difference (*p*=0.071). The cell numbers in the RJ group showed no significant intragroup difference. Moreover, both groups showed significantly lower concentrations of M-CSF in the baseline than after four weeks (*p* < 0.001).

### 3.5. Effect of RJ Intake on Physical Condition

The evaluation of physical condition by questionnaires is summarized in Table 5. Fatigue was evaluated by CFS scores. The CFS scores significantly decreased after four weeks of intake in both groups but with no significant differences between the two groups. Sleep quality was determined using the PSQI-J. In the pre-post comparisons for both groups, participants demonstrated a significant improvement in global PSQI-J scores, while there was no significant difference between the two groups. Among the subscores, a significant difference was noted in sleep latency between the two groups in the baseline (*p*=0.009), while intragroup sleep disturbance in the RJ group showed a significant improvement (*p*=0.039). In addition, the placebo group showed significantly lower sleep latency scores in comparison with the baseline after four weeks (*p* < 0.001). Anemia symptoms were assessed by the VAS. We found significant differences in shortness of breath and heart palpitations between the two groups before and after test food intake (*p*=0.024 and *p*=0.019, respectively). In the RJ group, difficulty getting up in the morning and nail brittleness, which is frequently caused by anemia, showed a significant improvement after four weeks compared with the baseline (*p*=0.007 and *p*=0.014, respectively). After four weeks of daily tablet intake, the placebo group displayed a lower sense of fatigue compared with the baseline (*p*=0.028).

### 3.6. Subgroup Analysis

A subgroup analysis was performed on the HSC count in peripheral blood, which is the primary end point. The number of HSCs in peripheral blood is affected by infections, such as colds. In this study, a daily log was kept to record clinical common cold-like symptoms during the intake period. Therefore, the subgroup analysis excluded 14 subjects (eight from the placebo and six from the RJ group), who complained of cold symptoms at baseline or within five days of the four-week study. Although hematocrit levels were significantly higher in the RJ group than those in the placebo group, it was within normal limits (*p*=0.045). We found no significant differences between the two groups in terms of age, BMI, HSC count, myeloid cell count, lymphoid cell count, and hematology parameters, except for hematocrit (Supplementary Table 2).

Table 6 presents the number of HSCs in peripheral blood by the subgroup analysis. The change in HSC count was significantly higher in the RJ group than that in the placebo group (*p*=0.042). After four weeks of daily tablet intake, the placebo group displayed a significantly lower cell count compared with the baseline, while the cell count in the RJ group showed no significant changes between baseline and post-administration after four weeks (*p*=0.047).

## 4. Discussion

To the best of our knowledge, this is the first study to explore the effects of RJ intake on human stem cells as indicated by the number of HSCs in peripheral blood. Our findings reveals that RJ increases HSCs in peripheral blood compared to placebo (Tables 2 and 6). In addition, the RJ group presented a significant improvement in blood pressure related to chronic vascular diseases, such as arteriosclerosis (Table 4).

Complementary and alternative medicine (CAM) is usually defined as various health care and medical practices independent of western medicine [21]. Currently, CAM, such as the Qingre Yiqi method with oral hypoglycemic drugs and pomegranate seed powder, has been utilized in type 2 diabetes mellitus, which is one of the chronic diseases [22, 23]. Furthermore, the previous study demonstrated that alkaloid berberine has been more effective in treating positive and negative symptoms of schizophrenia [24]. Also, RJ has been traditionally used as apitherapy, which is a part of CAM [25, 26]. Recent studies suggested that RJ administration resulted in effects on antioxidant, anti-inflammatory, and hypolipidemic activities in overweight adults [27], alleviated menopausal symptoms [11] and improved tear volume in patients with dry eye [28]. Globally, the rate of CAM application has approximately reached 9.8%–76.0% [29]. Hence, many countries and nationalities favor it due to its natural, convenient, and affordable properties [21].

Most of the HSCs targeted in this study were located in the bone marrow. The steady state of HSCs in peripheral blood is low, and they are mobilized from the bone marrow to peripheral blood when the body is in a critical condition, such as with infection [5]. Elderly individuals cannot efficiently mobilize HSCs into peripheral blood, even during a crisis state, and present a reduction in circulating HSC numbers [7]. The aged hematopoietic system as described above is considered to be associated with chronic diseases such as age-related immune disorders and anemia [8]. Also, reduced HPCs in peripheral blood were observed in children with chronic neutropenia who showed a higher incidence of severe infections and delayed self-resolution [16]. In this study, participants with a low number of HSCs in peripheral blood and an impaired immune system may have been preferentially selected. Therefore, higher HSC populations associated with RJ supplementation compared with those in placebo supplementation could alleviate age-associated symptoms such as immune decline partly due to a decrease in these cells. Moreover, a bacterial infection causes the mobilization of these cells from bone marrow [30]. Therefore, we can infer that the subjects who complained of cold symptoms had a higher number of HSCs in the peripheral blood than that in the normal state. Therefore, the effect of RJ on the HSC count can be easily explained, except for these subjects in the subgroup analysis.

To explain the mechanism of increasing HSC count in peripheral blood associated with RJ intake, we focused on CXCL12 secreted by osteoblasts in terms of mobilization from bone marrow. The CXCL12 receptor, C-X-C chemokine receptor type 4 (CXCR4), is located on the surface of HSCs and immune cells, and mobilization is controlled by the interaction of CXCL12 and CXCR4 [31, 32]. Both groups showed a significant increase after four weeks compared with the baseline, and the effect of RJ could not be confirmed. As another mechanism for mobilization to peripheral blood, neuropeptide Y secretion from the sympathetic nervous system is caused by activation of MMP-9 via the Y1 receptor on osteoblasts [33]. A previous study suggested that RJ alleviates autonomic nervous disorders and promotes the proliferation of mouse osteoblast-like cells [34, 35]. Thus, we could clarify the effect of RJ intake on HSC proliferation by conducting tests focusing on osteoblasts, which are also an HSC niche, and sympathetic nerves related to the autonomic nervous system.

Blood pressure is associated with various factors such as nitric oxide, reactive oxygen species, angiotensin-converting enzyme (ACE) activity, and stem cells like HSCs or EPCs [6, 36]. The low efficiency of circulating vascular EPCs causes vascular endothelial dysfunction, that is related to hypertension [37]. Therefore, we evaluated the effects of RJ on blood pressure and EPCs population in peripheral blood. Consequently, RJ significantly improved blood pressure compared to the placebo. However, the changes in the number of EPCs throughout the intervention were similar between them (Table 4). Several peptides in RJ inhibit ACE activity and RJ induces vasorelaxation through nitric oxide production from the vascular endothelium related to improvement of blood pressure in hypertensive rodents and humans, previously reported [38–41]. On the other hand, RJ consumption did not alter blood pressure in healthy human subjects, although it improved vascular endothelial function as assessed using RH-PAT [10]. These results suggested that the antihypertensive effect of RJ is still controversial and might involve multiple mechanisms. Further investigation is needed regarding the effect of RJ on blood pressure and various factor such as ACE activity, nitric oxide, and circulating EPCs.

Our study had some limitations. First, the sample size was insufficient to detect a small difference in peripheral HSC count. Second, the study was conducted during the season with the highest pollen count of the year in Japan, which induced allergic symptoms such as hay fever [42]. Although the influence of daily life and season on HSC count in peripheral blood is not known, the number of HPCs increased at the peak pollen count in Sweden, and eosinophils contained in the HPCs accumulated in tissues such as the airways, causing an allergic reaction [43]. Third, we could not assess the functional aspects of HSCs. Previous reports suggested that self-renewal, regeneration capacity, homing ability, and lymphoid-based differentiation of HSCs are reduced with aging [44]. Thus, further investigation of the effect of RJ on HSC function is needed.

## 5. Conclusions

In summary, since RJ intake affected the number of HSCs in peripheral blood, it may influence stem cell function. In addition, RJ could alleviate the age-associated immune decline partly due to decreasing HSCs and may contribute to healthy life expectancy. The effects on stem cells found throughout the body, including mesenchymal and neural stem cells, should be investigated to elucidate the various health-promoting effects of RJ.

## Figures and Tables

**Table 1:** Baseline characteristics of the study population.

	Placebo (mean ± SD) *n* = 42	RJ (mean ± SD) *n* = 44	*p* value
Age, years	55.2 ± 7.7	55.3 ± 9.0	0.954
Gender			
Female	33 (78.6%)	35 (79.5%)	0.912
Male	9 (21.4%)	9 (20.5%)	
BMI, kg/m^2^	21.9 ± 2.7	22.0 ± 2.5	0.934

*Stem cells*			
Hematopoietic stem cells, count/mL	620.7 ± 286.5	619.5 ± 263.1	0.983

*Hematology parameters*			
White blood cells, cells/*µ*L	5343.8 ± 1069.2	5462.2 ± 1331.8	0.561
Red blood cells, ×10^4^ cells/*µ*L	443.5 ± 44.9	448.6 ± 43.7	0.595
Hemoglobin, g/dL	13.3 ± 1.6	13.8 ± 1.4	0.112
Hematocrit, %	40.5 ± 4.1	42.1 ± 3.6	0.050
Mean corpuscular volume, fL	91.4 ± 5.5	94.1 ± 4.3	0.013
Mean corpuscular hemoglobin, pg	29.9 ± 2.4	30.8 ± 1.5	0.056
Mean corpuscular hemoglobin concentration, %	32.7 ± 1.2	32.7 ± 0.9	0.967
Platelet, ×10^4^ cells/*µ*L	25.8 ± 5.6	24.8 ± 6.3	0.438

*Myeloid cells*			
Basophil, %	0.5 ± 0.3	0.6 ± 0.3	0.255
Eosinophil, %	2.5 ± 2.1	2.8 ± 2.7	0.632
Monocyte, %	5.1 ± 1.0	5.1 ± 1.3	0.962
Neutrophil, %	59.5 ± 9.6	58.1 ± 8.4	0.475

*Lymphoid cells ※*			
Lymphocyte, count/*µ*L	1286.6 ± 351.6	1369.1 ± 418.0	0.360
B cell, count/*µ*L	166.5 ± 95.9	168.9 ± 90.1	0.910
NK cells, count/*µ*L	138.23 ± 104.1	138.0 ± 74.0	0.993
CD3^+^ T cells, count/*µ*L	598.0 ± 255.0	712.9 ± 259.9	0.057
CD4^+^ T cells, count/*µ*L	420.3 ± 189.0	509.2 ± 210.3	0.059
CD8^+^ T cells, count/*µ*L	151.0 ± 77.2	172.1 ± 72.1	0.226
CD8^+^CD28^+^ T cells, count/*µ*L	99.9 ± 54.5	115.0 ± 50.7	0.217

Values are mean ± SD. ※ 11 samples (six from placebo and five from RJ group) could not be measured due to hemolysis of red blood cells. *p* values compared with placebo by student's *t*-test. Gender was compared using the chi-square test. BMI, body mass index.

**Table 2:** The effects of RJ on hematopoietic stem cell count in peripheral blood.

Parameters		Placebo (mean ± SD) *n* = 42	RJ (mean ± SD) *n* = 42	*p*value student's *t*-test
Hematopoietic stem cells, count/mL	Baseline	620.7 ± 286.5	619.5 ± 263.1	0.983
	Week4	545.1 ± 217.8	642.7 ± 262.2	0.065
	Change	−75.6 ± 258.8	23.2 ± 295.1	0.103
	*p* value paired *t*-test	0.065	0.605	

Values are mean ± SD. *p* values compared with baseline by paired *t*-test or placebo using student's *t*-test.

**Table 3:** The effects of RJ on CXCL12 in serum.

Parameters		Placebo (mean ± SD) *n* = 42	RJ (mean ± SD) *n* = 44	*p*value student's *t*-test
CXCL12, pg/mL	Baseline	1429.3 ± 343.9	1336.0 ± 297.7	0.182
	Week4	1589.4 ± 341.3	1475.3 ± 351.4	0.130
	Change	160.2 ± 212.0	139.2 ± 218.5	0.653
	*p* value paired *t*-test	<0.001	<0.001	

Values are mean ± SD. *p* values compared with baseline by paired *t*-test or placebo using student's *t*-test.

**Table 4:** The effects of RJ on vascular endothelial function, blood pressure, and pulse rate.

Parameters		Placebo (mean ± SD) *n* = 42	RJ (mean ± SD) *n* = 44	*p*value student's *t*-test
*Vascular endothelial function*				
Endothelial progenitor cells, count/mL	Baseline	656.0 ± 330.3	651.1 ± 282.0	0.941
	Week4	559.3 ± 241.9	617.8 ± 258.8	0.282
	Change	−96.7 ± 338.2	−33.3 ± 299.1	0.359
	*p* value paired *t*-test	0.071	0.465	

M-CSF, pg/mL	Baseline	5.1 ± 1.6	5.1 ± 1.6	0.968
	Week4	4.1 ± 1.2	4.9 ± 1.4	0.311
	Change	−1.0 ± 1.2	−0.7 ± 0.9	0.180
	*p* value paired *t*-test	<0.001	<0.001	

*Blood pressure*				
Systolic, mm·Hg	Baseline	122.0 ± 16.3	123.6 ± 20.2	0.690
	Week4	121.0 ± 18.9	118.0 ± 17.4	0.446
	Change	−1.0 ± 11.5	−5.6 ± 10.4	0.055
	*p* value paired *t*-test	0.593	<0.001	

Diastolic, mm·Hg	Baseline	69.0 ± 10.4	69.7 ± 13.8	0.791
	Week4	69.6 ± 11.6	67.3 ± 13.0	0.372
	Change	0.6 ± 6.9	−2.5 ± 6.3	0.032
	*p* value paired *t*-test	0.562	<0.001	

*Pulse rate*				
Pulse rate, bpm	Baseline	73.9 ± 7.1	74.3 ± 9.8	0.795
	Week4	75.9 ± 8.7	75.4 ± 10.8	0.707
	Change	2.0 ± 6.9	1.1 ± 6.5	0.372
	*p* value paired *t*-test	0.024	0.232	

Values are mean ± SD. *p* values compared with baseline by paired *t*-test or placebo using student's *t*-test.

**Table 5:** .The effects of RJ questionnaire evaluation.

Parameters		Placebo (mean ± SD) *n* = 42	RJ (mean ± SD) *n* = 44	*p* value
*Chalder fatigue scale*				
Total	Baseline	16.5 ± 7.3	17.4 ± 8.0	0.511
	Week4	11.6 ± 5.9	11.7 ± 7.0	0.768
	Change	−4.9 ± 6.6	−5.7 ± 6.1	0.726
	*p* value Wilcoxon signed-rank test	<0.001	<0.001	

Physical	Baseline	9.5 ± 4.0	10.0 ± 5.1	0.639
	Week4	6.6 ± 3.6	6.6 ± 4.5	0.991
	Change	−2.9 ± 3.8	−3.5 ± 4.2	0.882
	*p* value Wilcoxon signed-rank test	<0.001	<0.001	

Mental	Baseline	7.0 ± 3.8	7.4 ± 3.4	0.624
	Week4	5.0 ± 2.7	5.2 ± 3.0	0.642
	Change	−2.0 ± 3.1	−2.2 ± 2.4	0.560
	*p* value Wilcoxon signed-rank test	<0.001	<0.001	

*Pittsburgh Sleep Quality Index*				
PSQI global score	Baseline	6.0 ± 2.0	5.8 ± 1.7	0.751
	Week4	5.2 ± 1.8	4.9 ± 1.9	0.539
	Change	−0.8 ± 1.6	−0.9 ± 1.9	0.653
	*p* value Wilcoxon signed-rank test	0.003	<0.001	

C1 sleep quality	Baseline	1.4 ± 0.6	1.3 ± 0.6	0.465
	Week4	1.3 ± 0.5	1.1 ± 0.5	0.078
	Change	−0.1 ± 0.6	−0.2 ± 0.6	0.577
	*p* value Wilcoxon signed-rank test	0.332	0.035	

C2 sleep latency	Baseline	1.4 ± 0.5	1.2 ± 0.4	0.009
	Week4	1.1 ± 0.4	1.1 ± 0.3	0.516
	Change	−0.3 ± 0.5	−0.1 ± 0.4	0.027
	*p* value Wilcoxon signed-rank test	<0.001	0.453	

C3 sleep duration	Baseline	1.4 ± 0.9	1.5 ± 0.7	1.000
	Week4	1.3 ± 0.8	1.4 ± 0.8	0.715
	Change	−0.1 ± 0.5	−0.1 ± 0.6	0.706
	*p* value Wilcoxon signed-rank test	0.146	0.424	

C4 sleep efficiency	Baseline	0.1 ± 0.5	0.3 ± 0.6	0.211
	Week4	0.1 ± 0.6	0.2 ± 0.5	0.157
	Change	0.0 ± 0.6	−0.1 ± 0.7	0.974
	*p* value Wilcoxon signed-rank test	0.750	0.547	

C5 sleep disturbance	Baseline	1.0 ± 0.4	0.8 ± 0.4	0.170
	Week4	0.9 ± 0.4	0.7 ± 0.5	0.057
	Change	−0.1 ± 0.5	−0.2 ± 0.5	0.590
	*p* value Wilcoxon signed-rank test	0.180	0.039	

C6 sleep medication	Baseline	0.1 ± 0.3	0.1 ± 0.2	0.673
	Week4	0.1 ± 0.2	0.0 ± 0.0	0.236
	Change	0.0 ± 0.3	−0.1 ± 0.2	1.000
	*p* value Wilcoxon signed-rank test	1.000	0.500	

C7 daytime dysfunction	Baseline	0.6 ± 0.6	0.7 ± 0.6	0.346
	Week4	0.5 ± 0.7	0.5 ± 0.7	0.809
	Change	−0.1 ± 0.7	−0.2 ± 0.6	0.509
	*p* value Wilcoxon signed-rank test	0.503	0.076	

*VAS of anemia symptoms*				
Vertigo	Baseline	4.2 ± 7.6	6.3 ± 13.2	0.360
	Week4	7.4 ± 18.5	5.3 ± 12.25	0.538
	Change	3.2 ± 16.4	−1.0 ± 10.7	0.157
	*p* value paired *t*-test	0.210	0.529	

Blackout	Baseline	2.1 ± 4.1	3.3 ± 10.6	0.500
	Week4	2.8 ± 6.1	1.9 ± 3.3	0.402
	Change	0.7 ± 5.7	−1.3 ± 11.4	0.296
	*p* value paired *t*-test	0.410	0.445	

Dizziness	Baseline	10.0 ± 11.1	16.1 ± 20.5	0.092
	Week4	10.0 ± 18.3	11.8 ± 15.4	0.619
	Change	0.0 ± 19.4	−4.3 ± 20.2	0.321
	*p* value paired *t*-test	1.000	0.168	

Difficulty getting up in the morning	Baseline	33.9 ± 29.8	41.0 ± 35.8	0.325
	Week4	26.3 ± 31.1	30.2 ± 34.9	0.587
	Change	−7.8 ± 29.9	−10.7 ± 25.1	0.596
	*p* value paired *t*-test	0.108	0.007	

Shortness of breath and heart palpitations	Baseline	13.5 ± 15.8	24.8 ± 27.7	0.024
	Week4	12.6 ± 16.3	23.4 ± 24.5	0.019
	Change	−0.9 ± 11.8	−1.3 ± 21.2	0.902
	*p* value paired *t*-test	0.632	0.677	

Nail brittleness	Baseline	19.2 ± 26.2	27.1 ± 32.2	0.216
	Week4	15.1 ± 24.1	19.9 ± 30.9	0.431
	Change	−4.1 ± 20.1	−7.2 ± 18.7	0.453
	*p* value paired *t*-test	0.200	0.014	

Sense of fatigue	Baseline	26.9 ± 24.6	29.1 ± 24.6	0.672
	Week4	17.7 ± 21.4	22.6 ± 21.7	0.300
	Change	−9.2 ± 26.1	−6.6 ± 23.7	0.630
	*p* value paired *t*-test	0.028	0.073	

Values are mean ± SD. The score of CFS and PSQI-J were compared using a Wilcoxon rank-sum test between the two groups. Differences between baseline and week 4 were investigated using a Wilcoxon signed-rank test. For the anemia symptoms, student's *t*-test was conducted to evaluated the differences between the two groups and paired *t*-test was used for statistical analysis of the intragroup.

**Table 6:** The effects of RJ on hematopoietic stem cell count in peripheral blood (subgroup analysis).

Parameters		Placebo (mean ± SD) *n* = 34	RJ (mean ± SD) *n* = 39	*p*value student's *t*-test
Hematopoietic stem cells, count/mL	Baseline	609.2 ± 289.8	593.2 ± 224.6	0.745
	Week4	528.3 ± 221.1	638.3 ± 269.2	0.075
	Change	−80.9 ± 228.5	45.1 ± 227.5	0.042
	*p* value paired *t*-test	0.047	0.330	

Values are mean ± SD. *p* values compared with baseline by paired *t*-test or placebo using student's *t*-test.

## Data Availability

The data used to support the findings of this study have been deposited in a private folder at Yamada Bee Company, Inc.

## Supplementary Materials

Supplementary Materials Supplementary Figure 1. Overview of the gating strategy for hematopoietic stem cells and endothelial progenitor cells. Supplementary Figure 2. Overview of the gating strategy for lymphoid cells. Supplementary Figure 3. List of questions of anemia symptoms assessed with 100-mm VAS. Supplementary Figure 4. Correlation between hematopoietic stem cell count in peripheral blood and age or BMI. Supplementary Table 1. All antibodies with clone ID and fluorochrome conjugate. Supplementary Table 2. Baseline characteristics of the study population (subgroup analysis).
