# Supplementary material for: Royal Jelly Increases Hematopoietic Stem Cells in Peripheral Blood: A Double-Blind, Placebo-Controlled, Randomized Trial in Healthy Subjects
Source: Evid Based Complement Alternat Med. 2023 Jan 13;2023:7665515. doi: 10.1155/2023/7665515 (PMC9859695; doi:10.1155/2023/7665515)
Supplement: Supplementary Materials — Supplementary Figure 1. Overview of the gating strategy for hematopoietic stem cells and endothelial progenitor cells. Supplementary Figure 2. Overview of the gating strategy for lymphoid cells. Supplementary Figure 3. List of questions of anemia symptoms assessed with 100-mm VAS. Supplementary Figure 4. Correlation between hematopoietic stem cell count in peripheral blood and age or BMI. Supplementary Table 1. All antibodies with clone ID and fluorochrome conjugate. Supplementary Table 2. Baseline characteristics of the study population (subgroup analysis). [file 7665515.f1.docx]

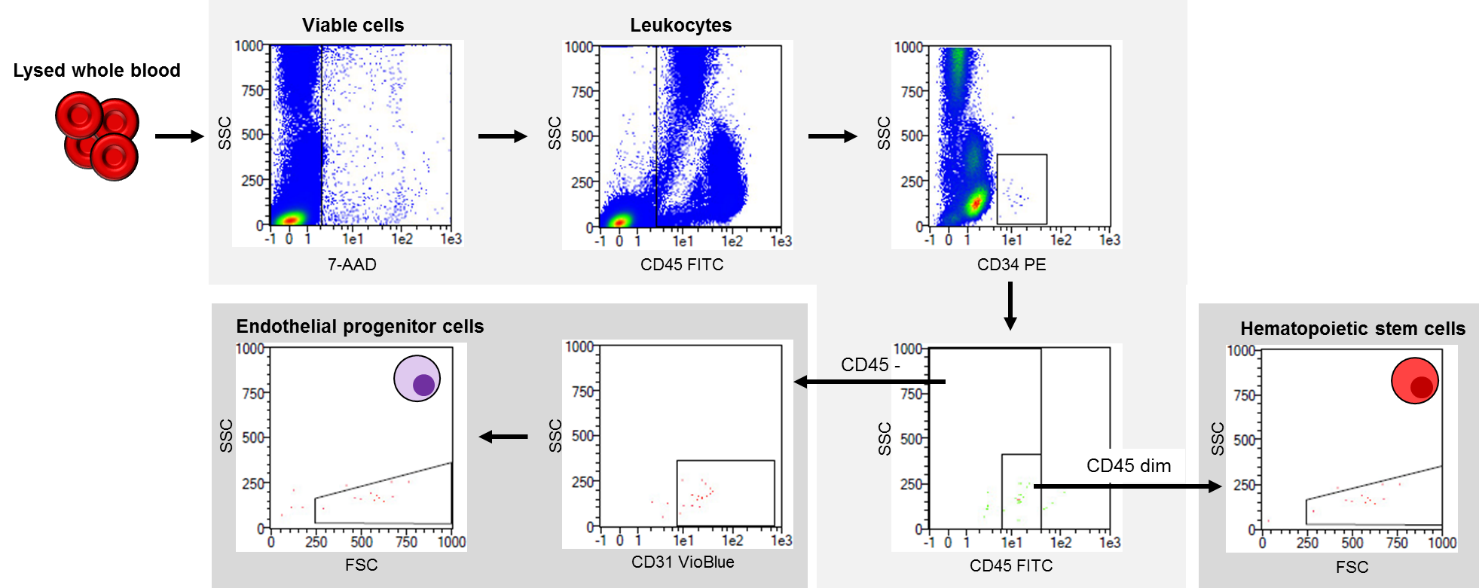


**Supplementary Figure 1. Overview of the gating strategy for hematopoietic stem cells and endothelial progenitor cells**

The data file of the stained lysed whole blood was analyzed as follows: exclusion of dead and apoptotic cells (7-AAD versus sideward scatter integral); gating of CD45^+^ leukocytes (anti-CD45 versus sideward scatter integral)—gated CD45^+^ leukocytes were used to discriminate undifferentiated stage of CD34^+^ stem cells (anti-CD34 versus sideward scatter integral); gating of CD45dim CD34^+^ stem cells (anti-CD45 versus sideward scatter integral)—gated CD34^+^CD45dim stem cells were used for the identification of hematopoietic stem cells (forward scatter integral versus sideward scatter integral) and the counted hematopoietic stem cell events for calculating the absolute cell number in whole blood; gated CD34^+^ stem cells were also used to distinguish CD34^+^CD45^−^ stem cells (anti-CD45 versus sideward scatter integral); gating of CD31^+^CD34^+^CD45^−^ cells (anti-CD31 versus sideward scatter integral)—gated CD31^+^CD34^+^CD45^−^ cells were used for the identification of endothelial progenitor cells (forward scatter integral versus sideward scatter integral) and the counted hematopoietic stem cell events for calculating the absolute cell number in whole blood.


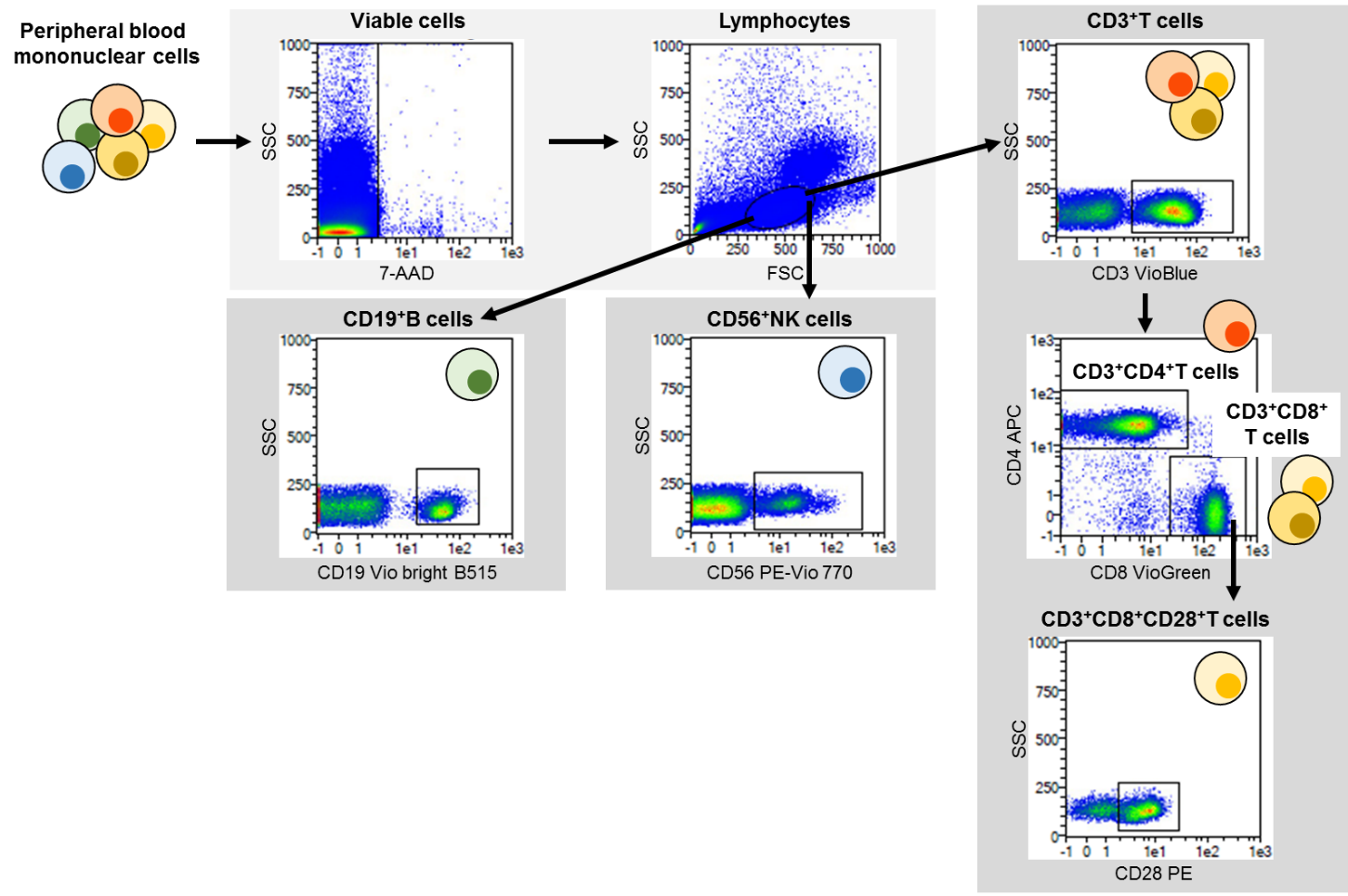


**Supplementary Figure 2. Overview of the gating strategy for lymphoid cells**

The data file of the stained peripheral blood mononuclear cells was analyzed as follows: exclusion of dead and apoptotic cells (7-AAD versus sideward scatter integral); gating of lymphocytes (forward scatter integral versus sideward scatter integral)—the lymphocytes were used as the reference for calculating the absolute cell number of indicated populations in whole blood; gating of CD56^+^NK cells (anti-CD56 versus sideward scatter integral); gating of CD3^+^T cells (anti-CD3 versus sideward scatter integral)—gated CD3^+^T cells were used for the identification of CD3^+^CD4^+^T cells and CD3^+^CD8^+^T cells (anti-CD8 versus anti-CD4)—gated CD3^+^CD8^+^T cells were further subdivided into CD3^+^CD8^+^CD28^+^T cells (anti-CD28 versus sideward scatter integral), and gated lymphocytes were used for the identification of the B-cell population (anti-CD19 versus sideward scatter integral).


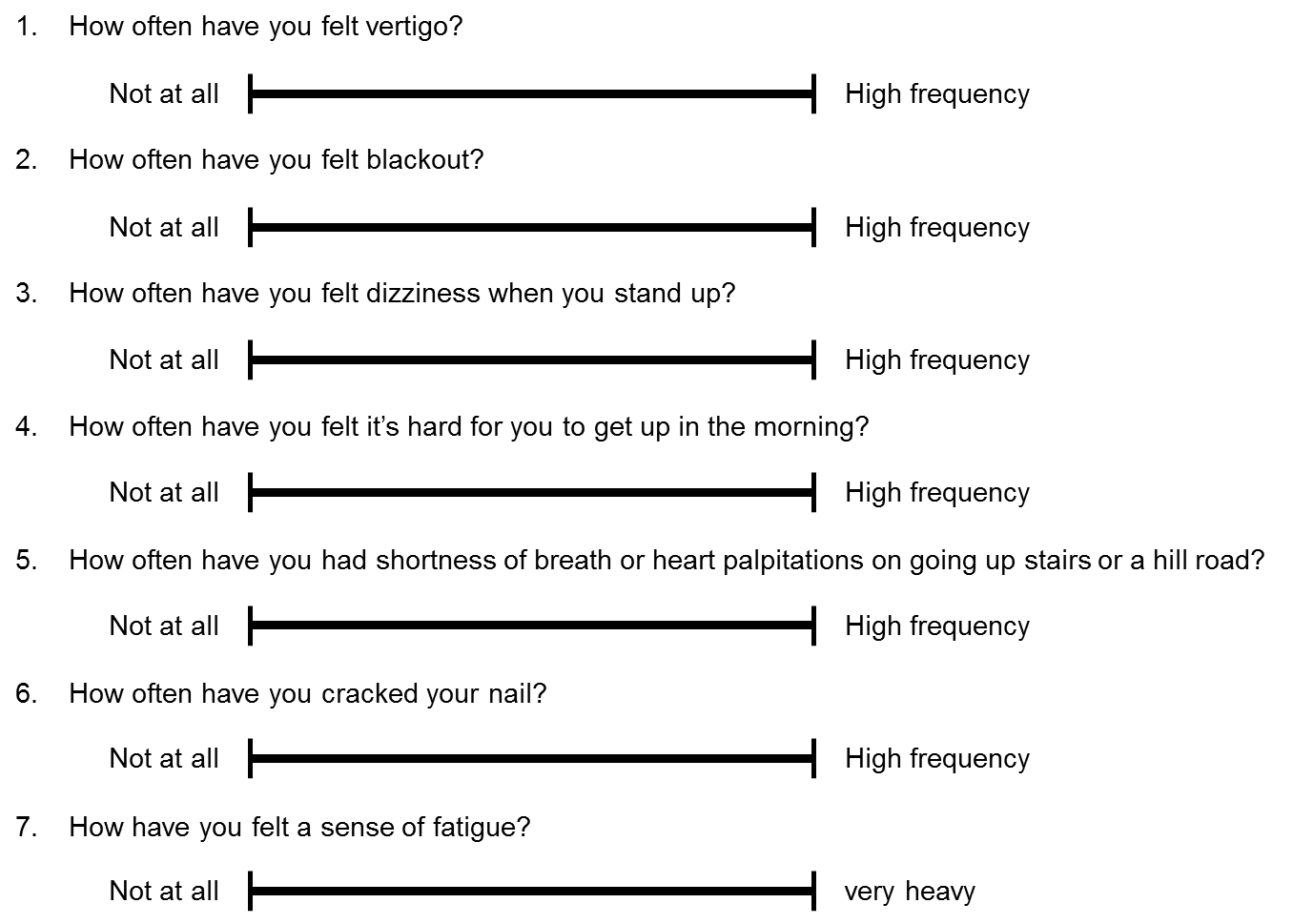


**Supplementary Figure 3. List of questions of anemia symptoms assessed with a 100-mm VAS**


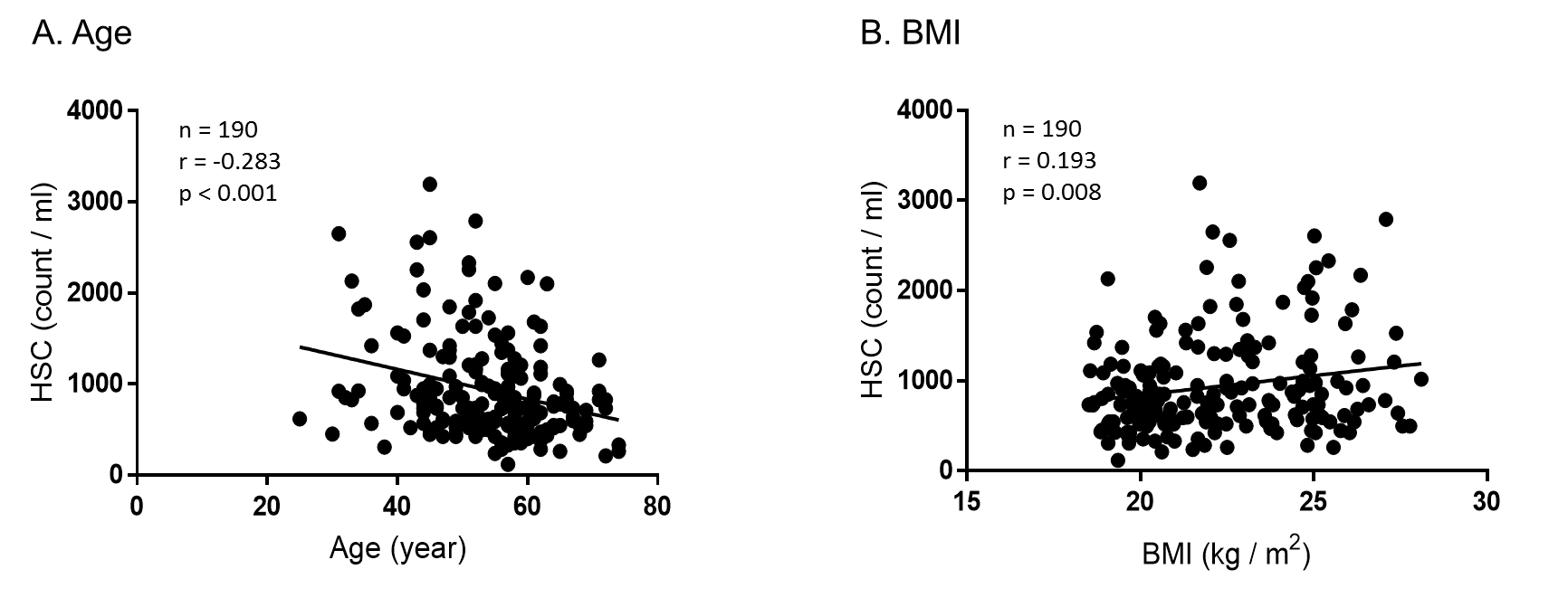


**Supplementary Figure 4. Correlation between hematopoietic stem cell count in peripheral blood and age or BMI**

(A) Hematopoietic stem cell count in peripheral blood plotted against age. (B) Hematopoietic stem cells count in peripheral blood plotted against BMI. r is the Pearson correlation coefficient between hematopoietic stem cell count in peripheral blood and age or BMI. BMI, body mass index; HSC, hematopoietic stem cell.

**Supplementary Table 1. All antibodies with clone ID and fluorochrome conjugate**

| Antibody | Source | Identifier |
| --- | --- | --- |
| Anti-human CD45 FITC/CD34 PE (Clone: 2D1, 8G12) | BD Biosciences | Cat#: 341071 |
| CD3 antibody, anti-human, VioBlue®, REAfinity™ (Clone: REA613) | Miltenyi Biotec | Cat#: 130-114-519 |
| CD4 antibody, anti-human, APC, REAfinity™ (Clone: REA623) | Miltenyi Biotec | Cat#: 130-113-222 |
| CD8 antibody, anti-human, VioGreen™, REAfinity™ (Clone: REA734) | Miltenyi Biotec | Cat#: 130-110-684 |
| CD19 antibody, anti-human, Vio® Bright B515, REAfinity™ (Clone: REA675) | Miltenyi Biotec | Cat#: 130-113-650 |
| CD28 antibody, anti-human, PE, REAfinity™ (Clone: REA612) | Miltenyi Biotec | Cat#: 130-123-782 |
| CD31 antibody, anti-human, VioBlue®, REAfinity™ (Clone: REA730) | Miltenyi Biotec | Cat#: 130-110-674 |
| CD56 antibody, anti-human, PE-Vio® 770, REAfinity™ (Clone: REA196) | Miltenyi Biotec | Cat#: 130-113-874 |
| 7-AAD Staining Solution | Miltenyi Biotec | Cat#: 130-113-313 |

**Supplementary Table 2. Baseline characteristics of the study population (subgroup analysis)**

|  | Placebo (*n* = 34) | RJ  (*n* = 38) | *P* value |
| --- | --- | --- | --- |
| Age, years | 55.0 ± 7.7 | 55.8 ± 8.5 | 0.661 |
| Gender |  |  |  |
| Female | 27 (79.4%) | 30 (78.9%) | 0.961 |
| Male | 7 (20.6%) | 8 (21.1%) |  |
| BMI, kg/m^2^ | 21.8 ± 2.6 | 22.0 ± 2.5 | 0.759 |
|  |  |  |  |
| Stem cells | | | |
| Hematopoietic stem cells, count/mL | 609.2 ± 289.8 | 589.3 ± 226.2 | 0.745 |
|  |  |  |  |
| Hematology parameters | | | |
| White blood cells, cells/µL | 5275.6 ± 1122.6 | 5470.8 ± 1346.0 | 0.509 |
| Red blood cells, ×10^4^ cells/µL | 440.2 ± 44.9 | 450.9 ± 44.9 | 0.313 |
| Hemoglobin, g/dL | 13.2 ± 1.6 | 13.8 ± 1.4 | 0.081 |
| Hematocrit, % | 40.2 ± 4.1 | 42.1 ± 3.7 | 0.045 |
| Mean corpuscular volume, fL | 91.6± 6.0 | 93.6 ± 4.0 | 0.096 |
| Mean corpuscular hemoglobin, pg | 29.9 ± 2.6 | 30.6 ± 1.5 | 0.164 |
| Mean corpuscular hemoglobin  concentration, % | 32.6 ± 1.2 | 32.7 ± 0.9 | 0.745 |
| Platelet, ×10^4^ cells/µL | 25.4 ± 5.5 | 24.0 ± 5.2 | 0.259 |
|  |  |  |  |
| Myeloid cells | | | |
| Basophil, % | 0.5 ± 0.3 | 0.6 ± 0.3 | 0.295 |
| Eosinophil, % | 2.3 ± 2.1 | 2.8 ± 2.8 | 0.382 |
| Monocyte, % | 5.0 ± 1.0 | 5.0 ± 1.3 | 0.851 |
| Neutrophil, % | 59.7 ± 10.2 | 58.2 ± 8.7 | 0.522 |
|  |  |  |  |
| Lymphoid cells ※ | | | |
| Lymphocyte, count/µL | 1274.8 ± 374.1 | 1360.4 ± 426.4 | 0.412 |
| B cell, count/µL | 162.9 ± 102.5 | 163.3 ± 79.4 | 0.989 |
| NK cell, count/µL | 148.0 ± 112.5 | 138.1 ± 75.9 | 0.686 |
| CD3^+^ T cell, count/µL | 611.4 ± 228.1 | 713.1 ± 276.6 | 0.127 |
| CD4^+^ T cell, count/µL | 423.1 ± 169.7 | 515.0 ± 224.0 | 0.080 |
| CD8^+^ T cell, count/µL | 159.1 ± 70.5 | 168.8 ± 76.1 | 0.608 |
| CD8^+^CD28^+^ T cell, count/µL | 106.1 ± 50.7 | 108.7 ± 49.9 | 0.839 |

Values are mean ± SD. ※ 11 samples (six from placebo and five from RJ group) could not be measured due to hemolysis of red blood cells. *P* values compared with placebo by Student’s *t*-test. Gender were compared using the Chi-square test. BMI: body mass index.
